# Aptamer-guided DNA tetrahedron as a novel targeted drug delivery system for MUC1-expressing breast cancer cells *in vitro*

**DOI:** 10.18632/oncotarget.9431

**Published:** 2016-05-18

**Authors:** Bindong Dai, Yan Hu, JinHong Duan, Xian-Da Yang

**Affiliations:** ^1^ Institute of Basic Medical Sciences, Chinese Academy of Medical Sciences & Peking Union Medical College, Beijing, China

**Keywords:** MUC1, aptamer, DNA tetrahedron, targeted drug delivery, cancer

## Abstract

Mucin 1 (MUC1) is an important molecular target for cancer treatment because it is overexpressed in most adenocarcinomas. In this study, a new MUC1-targeted drug delivery system was assembled using an aptamer (Apt) that could recognize MUC1 and a DNA tetrahedron (Td) that could carry doxorubicin (Dox) within its DNA structure. The complex thus formed (Apt-Td) had an average size of 12.38 nm and was negatively charged. Similar to the MUC1 aptamer, the Apt-Td could preferentially bind with MUC1-positive MCF-7 breast cancer cells. A drug loading experiment revealed that each Apt-Td complex could carry approximately 25 Dox molecules. Moreover, Apt-Td selectively delivered Dox into the MUC1-positive breast cancer cells but reduced Dox uptake by the MUC1-negative control cells. Dox-loaded Apt-Td also induced a significantly higher cytotoxicity to the MUC1-positive cancer cells versus the MUC1-negative control cells *in vitro* (p<0.01). These results suggest that Apt-Td may potentially serve as a drug carrier in the targeted treatment of MUC1-expressing breast cancers.

## INTRODUCTION

Chemotherapy is the primary treatment for advanced metastatic breast cancer. However, conventional chemotherapy faces the major obstacle of systematic toxicity [1, 2]. Because most conventional anticancer agents cannot distinguish between normal and tumor cells, they produce side effects, such as nausea, hair loss, neutropenia, peripheral neuropathies, renal failure, encephalopathy, and cardiac toxicity [3–8]. These adverse reactions seriously limit the efficacy of chemotherapy to eliminate metastatic cancer cells because the drug dosage and treatment frequency are often curbed by patients' intolerance to treatment-associated side effects. As a result, it is often impossible for chemotherapy to eradicate all tumor cells within the body, leading to tumor recurrence and poor prognosis. Therefore, exploring new therapeutic strategies for advanced cancer is a matter of medical importance.

One strategy for overcoming the systemic toxicity of chemotherapy is targeted tumor therapy. Because a targeted drug delivery system can selectively guide therapeutics into tumor cells, the effective accumulation of anticancer agents occurs in the tumor, but not in normal tissue. Thus, treatment-related side effects are significantly reduced compared to conventional chemotherapy [9]. Park et al. demonstrated that HER2 antibody-guided liposomes loaded with doxorubicin markedly improved the therapeutic index with animal tumor models, both by increasing antitumor efficacy and by reducing systemic toxicity [10]. MacDiarmid et al. showed that EGFR-targeted minicells loaded with chemotherapeutics achieved efficacious tumor inhibition *in vivo* with decreased systemic toxicity [11]. Moreover, the FDA has approved two antibody–drug conjugates (ADCs) for cancer treatment (brentuximab vedotin and trastuzumab emtansine) [12, 13], and there are more than 30 clinical trials testing new ADCs for oncological applications [14]. Therefore, targeted tumor treatment not only enhances antitumor efficacy but is also a pivotal strategy for reducing the adverse reactions associated with conventional chemotherapy [15].

MUC1 has been recognized as an important molecular target for cancer treatment. It is a cell surface glycoprotein that is widely overexpressed in many types of adenocarcinomas, including cancers of the lung, colon, pancreas, stomach, ovary, and breast, the latter being the most common malignancy in women with millions of cases worldwide [16]. Prior studies have demonstrated that MUC1 in cancer cells is under glycosylated, exposing the protein backbone and increasing the proteinogeneic accessibility by ligands such as antibodies or aptamers [17–19]. This feature, together with the fact that MUC1 is overexpressed in most carcinoma cells, makes MUC1 an attractive therapeutic target. Several MUC1-binding ligands have been developed and utilized for targeted delivery of chemotherapeutics or phototoxin to MUC1-positive cancer cells *in vitro* [17, 20–22]. Owing to the technical difficulties, however, no MUC1-targeted drug delivery system has been developed to a stage ready for preclinical evaluation. Due to the potential of MUC1 to serve as a broad-spectrum target for cancer treatment, it is necessary to explore new MUC1-targeted drug-delivery system designs, to facilitate the development of pharmaceutically implementable targeted chemotherapy against MUC1-expressing tumors.

In this study, we designed a new MUC1-targeted drug delivery system using a MUC1 aptamer and a DNA Td. Aptamers are short, single-stranded oligonucleotides (DNA and RNA) that can form complicated three dimensional structures and bind with a target molecule with high specificity and affinity [23]. As tumor-targeting ligands, aptamers have certain advantages compared with antibodies, including a high capacity for penetrating solid tumors, low immunogenicity, high binding specificity, low production cost, and consistent quality among production batches [24, 25]. DNA Td holds some advantages as a potential drug carrier of the anticancer agent doxorubicin [26]. It can be conveniently self-assembled from four DNA single strands into a stable structure with a precisely controlled size and high production yield. Moreover, a DNA Td can load doxorubicin within its DNA strands and carry significantly more drug molecules than a free aptamer *per se* [26–28]. Furthermore, it is theoretically possible to link a tumor-targeting aptamer with a DNA tetrahedron using the principle of DNA complementary base pairing in a self-assembled manner, avoiding the catalyst-mediated chemical reactions that usually require complicated purification protocols with increased production cost. Thus far, however, there have been no reports in the literature on using an aptamer-guided DNA tetrahedron for targeted drug delivery to cancer cells. It is unclear whether a tumor-targeting aptamer can be assembled onto a tetrahedron via DNA complementary base pairing, and whether the complex thus formed can serve as a targeted drug delivery system. In this study, we attempted to construct the first aptamer-tetrahedron complex (Apt-Td) for the targeted delivery of doxorubicin to MUC1-positive cancer cells. The basic properties of the Apt-Td complex and its efficacy as a targeted drug delivery system were evaluated *in vitro*, using the MUC1-expressing MCF-7 breast cancer cell line as the model system. We here report that Apt-Td delivers doxorubicin to MUC1-positive breast cancer cells in a targeted manner.

## RESULTS

### Preparation of Apt-Td

**Figure 1:**
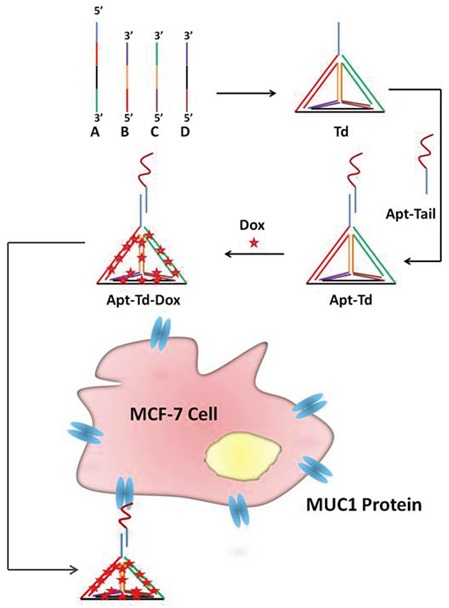
Schematic illustration of the design of the aptamer-modified DNA tetrahedron for selective delivery of doxorubicin to MUC1-positive breast cancer cells Four DNA single strands assembled into a DNA tetrahedron (Td) via DNA complementary base pairing. One of the four DNA strands was extended with a sticky end, which was exposed outside the Td. The MUC1 aptamer was also extended with a tail (Apt-tail), which could pair with the sticky end of the Td. The aptamer-Td complex thus formed (Apt-Td) was mixed with doxorubicin (Dox) to form the Apt-Td-Dox, which would bind with MCF-7 cancer cells for targeted drug delivery.

**Figure 2:**
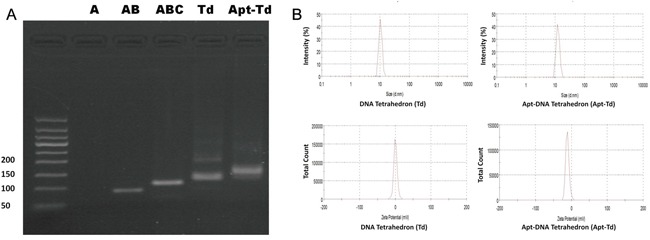
Formation and characterization of Td and Apt-Td **A.** Agarose gel electrophoresis was utilized to verify the assembling of the Td and Apt-Td. The four single strand DNA chains for Td were named A, B, C, and D, respectively. They were mixed into various combinations of A, AB, ABC, and ABCD (Td), respectively. The last lane showed the result of Td mixed with Apt-tail, for formation of Apt-Td. The result of strand A was not well visualized, presumably because the fluorescent dye could not stain single strand DNA well. **B.** Evaluation of particle size and zeta-potential of Td and Apt-Td with dynamic light scattering (DLS). Td had an average size of 10.4 nm and an average zeta-potential of −1.22 mV. Apt-Td had an average size of 12.38 nm and an average zeta-potential of −10.6 mV.

Apt-Td was assembled as illustrated in Figure 1. Previous work by Wang et al. has clearly demonstrated that the four DNA strands involved in this study can self-assemble into a DNA tetrahedron [29]. Here in this study, the original MUC1 aptamer was extended with an extra tail (Apt-tail) to serve as a sticky end. A complementary strand to the sticky end of the aptamer was extended from one of the four strands that composed the DNA Td. Thus, the Apt should be able to couple with the Td according to the principle of DNA complementary base pairing. Doxorubicin was later intercalated into the Apt-Td to complete the targeted drug carrier system. To evaluate whether these DNA strands could be assembled into one complex, electrophoresis was conducted. As shown in Figure 2A, the four single strands could indeed assemble into the structure of DNA Td as previously described [29]. Moreover, the Apt-tail coupled with the Td and formed a larger DNA structure, indicating that Apt-Td could be constructed following our method. To characterize the size and zeta-potential of these DNA nanostructures, a dynamic light scattering study was performed. The results showed that the average size of Td was 10.40 nm before coupling with the aptamers. The average Apt-Td size slightly increased to 12.38 nm, presumably because of the added structure of the aptamer. All of the above nanostructures were negatively charged. The average zeta-potentials of the Apt-Td and Td were −10.6 mV and −1.22 mV (Figure 2B), respectively.

### Affinity of MUC1 aptamer and Apt-Tail to MUC1-positive and MUC1-negative cell lines

**Figure 3:**
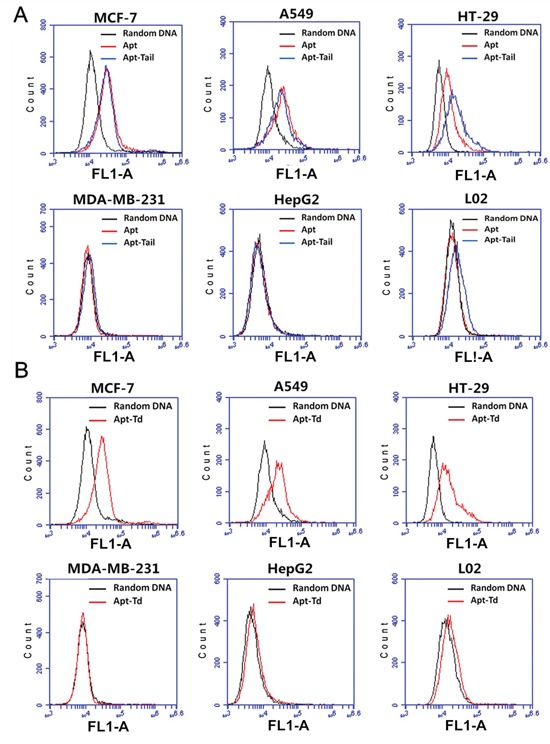
Evaluation of the binding of Apt, Apt-tail, or Apt-Td to MUC1-positive and MUC1-negative cells FAM-labeled random DNA, Apt, Apt-tail or Apt-Td were incubated with the cells for 30 minutes and washed. Flow cytometry was performed to evaluate the binding of Apt, Apt-tail **A.**, or Apt-Td **B.** to both MUC1-positive cells and MUC1-negative cells. The black curves represent the results of random DNA, the blue curves represent the results of the Apt-tail, and the red curves represent the results of Apt or Apt-Td.

The aptamer adopted in this study (S2.2) has been reported to bind to MUC1 with high affinity [20]. To test whether the aptamer could indeed differentiate between the MUC1-positive and the MUC1-negative cells, its binding with these two types of cells was evaluated by flow cytometry, using a random DNA pool as the control. Previous research has well established that the cell lines of MCF-7, A549 and HT-29 overexpress MUC1, and that the MDA-MB-231, HepG2 and L02 cells are MUC1-negative [17, 30–32]. The flow cytometric results (Figure 3A) revealed that random DNA generated some low-level binding to MUC1-positive cell lines and MUC1-negative cell lines, presumably because of the non-specific binding of DNA to these cells. Compared with random DNA, however, the Apt bound much more strongly to MUC1-positive cells, but not to MUC1-negative cells, suggesting that the Apt could indeed differentiate between the MUC1-positive and the MUC1-negative cells. To evaluate whether the Apt-tail, modified from the original Apt, could still differentiate between the MUC1-positive and the MUC1-negative cells, the Apt-tail was also incubated with the cells and evaluated by flow cytometry. The results showed that the Apt-tail also bound strongly to the MUC1-positive cells versus the MUC1-negative cells (Figure 3A), indicating that the Apt-tail largely retained the binding properties of the aptamer S2.2 and could be used as a ligand for targeting the MUC1 proteins.

### Apt-Td selectively bound to MUC1-positive cells

Although the above data demonstrated that both Apt and Apt-tail could recognize MUC1-positive cells, it was still unknown whether the assembled Apt-Td could also distinguish between the MUC1-positive and the MUC1-negative cells. To address this issue, FAM-labeled Apt-Td was incubated with the cells and evaluated by flow cytometry. The results showed that Apt-Td also demonstrated significantly higher binding to MUC1-positive cells versus MUC1-negative control cells (Figure 3B), indicating that Apt-Td retained the capacity to preferentially bind with the MUC1-positive cancer cells and thus may serve as a potential MUC1-targeting drug carrier.

### Drug-loading capacity of Apt-Td

**Figure 4:**
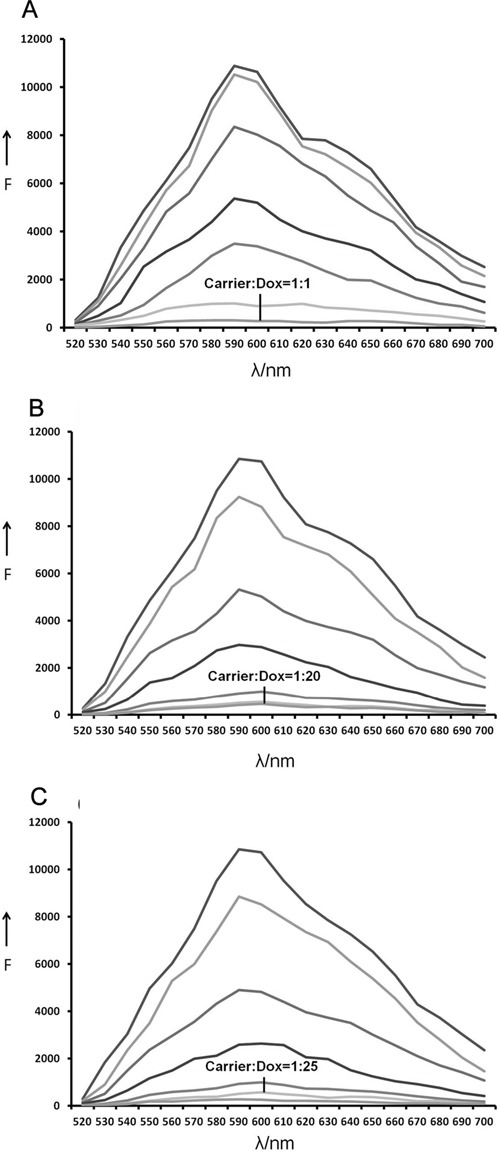
Estimation of the drug-loading capacities of Apt, Td and Apt-Td The fluorescence emitted by doxorubicin was quenched after the drug was intercalated into DNA. This phenomenon was used here to evaluate the drug loading capacity of the DNA structures. **A.** Fluorescence spectra of Dox solution mixed with increasing molar ratios of the MUC1 Apt (from top to bottom: 0, 0.0001, 0.005, 0.01, 0.05, 0.5, and 1), the fluorescence was quenched at the molar ratio 1:1. **B.** Fluorescence spectra of Dox solution mixed with increasing molar ratios of the Td (from top to bottom: 0, 0.0001, 0.005, 0.001, 0.01, 0.05, and 1), the fluorescence was quenched at the molar ratio 1:20. **C.** Fluorescence spectra of the doxorubicin solution mixed with increasing molar ratios of the Apt-Td (from top to bottom: 0, 0.0001, 0.005, 0.001, 0.01, 0.04, and 1), the fluorescence was quenched at the molar ratio 1:25.

For any drug delivery system, it is important to evaluate its drug loading capacity. Previous studies have demonstrated that doxorubicin can intercalate into DNA structures. Moreover, free doxorubicin has a red fluorescence that is quenched when the drug is inserted into DNA [27]. This phenomenon can be employed to estimate the amount of doxorubicin that has been absorbed by a given DNA structure [28]. In this study, Apt, Td, or Apt-Td were mixed with doxorubicin separately to from drug-loaded DNA structures of Apt-Dox, Td-Dox, and Apt-Td-Dox, respectively. To estimate the drug loading capacity of the DNA structures, Apt, Td, or Apt-Td were mixed with doxorubicin at increasing molar ratios, and analyzed by fluorescence spectroscopy. As shown in Figure 4, the fluorescence of doxorubicin was completely quenched when the molar ratio of DNA structure to doxorubicin increased to 1, 0.05, and 0.04, for Apt, Td, and Apt-Td, respectively. The results indicated that the Dox-loading capacity of Apt, Td, and Apt-Td was approximately 1, 20, and 25, respectively. In other words, each Td or Apt-Td could carry 20 times more doxorubicin than a free aptamer, making them superior drug carrier candidates. Therefore, in subsequent studies, Td and Apt-Td were further evaluated as doxorubicin carriers.

### Apt-Td selectively delivered Dox to MUC1-positive MCF-7 cells

**Figure 5:**
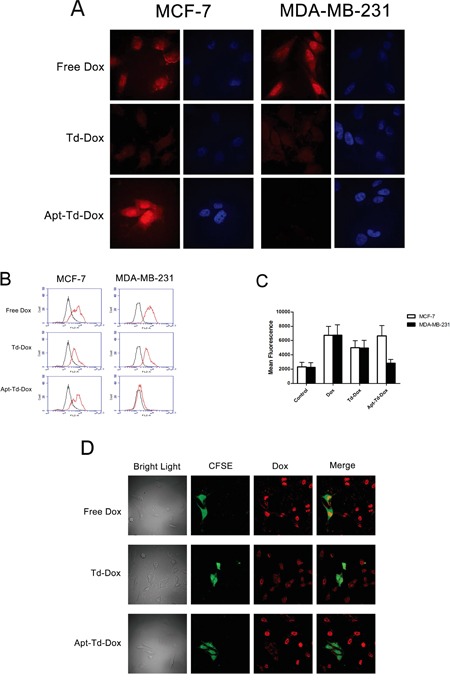
Evaluation of the cellular uptake of doxorubicin by MUC1-positive cells and MUC1-negative cells treated with Dox, Td-Dox, or Apt-Td-Dox **A.** Confocal scanning microscopic images of cells. Doxorubicin was loaded into Td and Apt-Td to form Td-Dox and Apt-Td-Dox. The MUC1-positvie cells and MUC1-negative cells were treated separately with free Dox, Td-Dox or Apt-Td-Dox for 1.5 h in PBS. Multiple scans through various levels of the cells were obtained. The central level scan went through the center of the cells and the nuclei were displayed. The Dox emitted a red fluorescence, which was largely observed in the nuclear region of the cells. The cytoplasm staining was relatively weak compared to that of the nuclear region. The nuclei were also stained blue with DAPI. **B.** Flow cytometric analysis of the cells treated with free Dox, Td-Dox, or Apt-Td-Dox for 1.5 hours. The black curves represent the untreated control cells, and the red curves indicate the cells treated with Dox, Td-Dox or Apt-Td-Dox. **C.** The value of mean fluorescent intensity obtained from the cytometic analysis of MCF-7 and MDA-MB-231 cells incubated with free Dox, Td-Dox, or Apt-Td-Dox. The mean fluorescence intensity was the average value of the fluorescence of the cells analyzed by flow cytometry, and calculated by the software associated with the FACS machine. **D.** Co-culture experiments for further evaluation of the targeting capability of Apt-Td-Dox. The bright light image contained both MCF-7 and MDA-MB-231 cells. A portion of these cells were MDA-MB-231 cells, which were stained by CFSE that emitted green fluorescence, while the MUC1-positive MCF-7 cells were not stained with CFSE. The cells were co-cultured in the same dish, treated with free Dox (with red fluorescence), Td-Dox, or Apt-Td-Dox, and evaluated by confocal microscopy.

The above studies on drug-loading capacity revealed that both Td and Apt-Td could carry a certain amount of doxorubicin. For targeted therapy, the key question was which drug carrier could increase the doxorubicin amount in MUC1-positive cells while simultaneously reducing the drug amount in MUC1-negative cells. To address this issue, free Dox, Td-Dox, or Apt-Td-Dox were incubated with MUC1-positive and the MUC1-negative cells separately. Confocal microscopy was utilized to evaluate the red fluorescence emitted by the doxorubicin within the cells. Multiple scans through various levels of the cells were obtained. The central level scan went through the center of the cells allowing the nuclei to be displayed (Figure 5A), clearly indicating that doxorubicin could be internalized into the cells, and mainly accumulated within the nuclei. When treated with free Dox, the drug accumulated in both the MUC1-positive and the MUC1-negative cells (Figure 5A, the upper panel), suggesting that doxorubicin *per se* could readily diffuse across the cell membrane and enter both types of cells. When treated with Td-Dox, however, the amounts of doxorubicin in both cell types were similar but significantly reduced (Figure 5A, the middle panel), presumably because there was a repulsive force between the negatively charged DNA Td and the cells that were also negatively charged [33]. When treated with Apt-Td-Dox, significantly more doxorubicin was observed in the MUC1-positive cancer cells compared with the MUC1-negative control cells (Figure 5A lower panel), indicating that a targeted-delivery of doxorubicin occurred *in vitro*.

To further evaluate whether Apt-Td-Dox could be selectively taken up by MUC1 positive cells, flow cytometry was also conducted to monitor the fluorescence generated by doxorubicin after incubating the two cell lines with free Dox, Td-Dox, or Apt-Td-Dox. For MUC1-positive cells, the fluorescent signals generated by free Dox or Apt-Td-Dox were similar (Figure 5B & 5C), whereas for MUC1-negative cells, the fluorescent signals generated by Apt-Td-Dox were remarkably lower than those generated by free Dox (Figure 5B & 5C). These results again indicated that Apt-Td-Dox could be selectively taken up by MUC1-positive cancer cells.

Co-culture experiment was also performed to evaluate the targeting specificity of Apt-Td-Dox. To distinguish different cell types, MUC1-negative cells (MDA-MB-231) were stained with CFSE that emitted a green fluorescence, while MUC1-positive cells (MCF-7) were not stained. The cells were co-cultured together, treated with free Dox (with red fluorescence) or Apt-Td-Dox, and evaluated with confocal microscope. As shown in Figure 5D, when treated with free Dox, red fluorescence from doxorubicin was strong in both cell types, indicating that free Dox could enter both the MUC1-positive and the MUC1-negative cells. When treated with Apt-Td-Dox, however, red fluorescence in MCF-7 cells was significantly stronger than that in MDA-MB-231 cells, indicating that Apt-Td-Dox primarily delivered Dox into the MUC1-positive cells, with limited cross-delivery to the MUC1-negative cells. Taken together, the microscopy and flow cytometry data indicated that the Apt-Td may serve as a carrier for targeted delivery of Dox to MUC1-positive cancer cells *in vitro.*

### Apt-Td-Dox induced targeted cytotoxicity against MUC1-positive cancer cells *in vitro*

**Figure 6:**
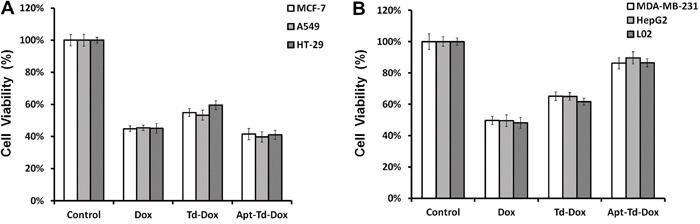
Apt-Td-Dox induced a targeted cytotoxicity against MUC1-positive cancer cells *in vitro* Free Dox, Td-Dox, or Apt-Td-Dox were incubated with MUC1-positive and MUC1-negative cells separately for two hours in PBS and washed. An MTS assay was used to evaluate the cell viability after further cultivation for 48 hours. The average cell viabilities for MUC1-positive cells (MCF-7, A549 and HT-29) **A.** and MUC1-negative cells (MDA-MB-231, HepG2 and L02) **B.** were presented (mean ± SD, n = 6). The cell viability of Dox, Td-Dox, and Apt-Td-Dox treated MCF-7 cells were 44.8%, 54.9%, and 35.4%, respectively. The cell viability of Dox, Td-Dox, and Apt-Td-Dox treated MDA-MB-231 cells were 47%, 65.1%, and 86.3%, respectively.

Although the above data demonstrated that Apt-Td could carry more doxorubicin into MUC1-positive cancer cells versus MUC1-negative control cells, it was still unknown whether Apt-Td-Dox would generate a MUC1-targeted cytotoxicity against the tumor cells. To investigate whether Apt-Td-Dox could indeed enhance the cytotoxicity against MUC1-positive cells and reduce toxicity to MUC1-negative control cells, the cells were incubated with free Dox, Td-Dox, or Apt-Td-Dox separately. Drug-induced cytotoxicity was subsequently evaluated with a standard MTS cell viability assay. As shown in Figure 6, free Dox yielded similar degrees of cytotoxicity to both MUC1-positive and MUC1-negative cells. Td-Dox also generated similar degrees of cytotoxicity in both types of cells, but at a reduced level compared to that induced by free Dox (Figure 6). Apt-Td-Dox, however, generated significantly greater cytotoxicity against the MUC1-positive cancer cells versus MUC1-negative control cells (Figure 6). Specifically, compared to free Dox, Apt-Td-Dox induced comparable cytotoxicity in MUC1-positive cells (P<0.05, Figure 6A) but significantly reduced the cytotoxicity to MUC1-negative control cells (P<0.01, Figure 6B). These data demonstrated that Apt-Td-Dox could generate MUC1-targeted cytotoxicity *in vitro* and that aptamer-modification markedly improved the performance of the DNA tetrahedron as a drug carrier for targeted therapy.

### Internalization time course and IC50 of Apt-Td-Dox

**Figure 7:**
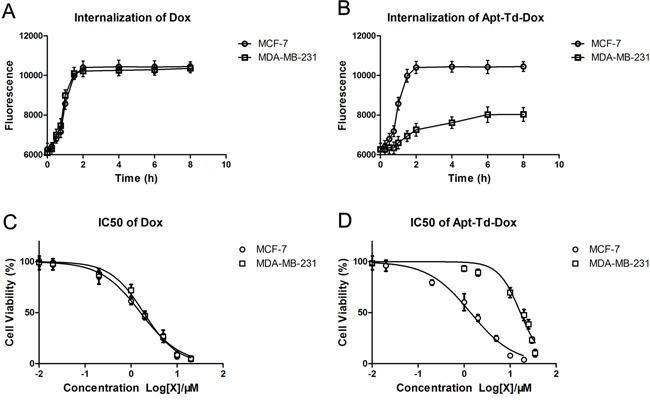
Internalization speed and IC50 of Apt-Td-Dox Free Dox **A.** or Apt-Td-Dox **B.** were incubated with MUC1-positive and MUC1-negative cells separately in PBS for 0.25, 0.5, 0.75, 1, 1.5, 2, 4, 6, and 8 hours, washed, and evaluated by flow cytometry to monitor the internalization of free Dox that emitted a red fluorescence. IC50 was evaluated by incubating various concentration of free Dox or Apt-Td-Dox with MUC1-positive and MUC1-negative cells in PBS for 2 hours. The cells were washed, and after cultivation for another 48 hours, analyzed by MTS assay for cell viability. The IC50 curves of free Dox **C.** or Apt-Td-Dox **D.** were generated by the software GraphPad Prism 5 (mean ± SD, n = 6).

To further explore the internalization speed of the Apt-Td-Dox, the time course of intracellular drug accumulation was evaluated. Free Dox or Apt-Td-Dox was incubated with the MCF-7 cells and MDA-MB-231 cells separately for various time durations. The cells were washed and evaluated by flow cytometry to monitor the mean fluorescence generated by doxorubicin. The results showed that free Dox entered both types of cells at similar speed (Figure 7A). However, Apt-Td-Dox entered the MUC1-positive MCF-7 cells at a higher speed vs. the MUC1-negative MDA-MB-231 cells (Figure 7B).

To further characterize the pharmacological property of Apt-Td-Dox, the IC50 was also evaluated. Free Dox and Apt-Td-Dox of increasing concentrations were incubated with MCF-7 or MDA-MB-231 cells, which were evaluated for cellular viability. The results showed that free Dox had a similar IC50 for both MCF-7 and MDA-MB-231 cells (Figure 7C), whereas Apt-Td-Dox had a significantly higher IC50 for the MUC1-negative cells vs. the MUC1-positive cells (16.72 μM vs. 1.381 μM, Figure 7D). The results again indicated that the Apt-Td-Dox selectively targeted the MUC1-positive cells *in vitro*.

## DISCUSSION

The primary goal of this study was to evaluate whether aptamer-guided DNA tetrahedrons could selectively deliver doxorubicin to MUC1-expressing breast cancer cells. The aptamer was coupled to the tetrahedron via DNA complementary base pairing in a self-assembled manner (Figure 1). The structure thus formed (Apt-Td) had an average size of 12.38 nm and was negatively charged (Figure 2). Similar to the free MUC1 aptamer, Apt-Td could differentiate between the MUC1-positive and the MUC1-negative cells (Figures 3). The drug-loading capacity of Apt-Td (25 per Apt-Td) was significantly higher than that of free aptamer (one per aptamer, Figure 4). Confocal microscopy and flow cytometry demonstrated that Apt-Td selectively delivered Dox into the MUC1-positive cancer cells (Figure 5). Moreover, Dox-loaded Apt-Td induced a significantly higher cytotoxicity to MUC1-positive cancer cells versus MUC1-negative control cells (Figure 6 & 7), indicating that Apt-Td achieved targeted drug delivery to MUC1-positive tumor cells *in vitro*.

The mechanism by which Apt-Td enters the MUC1-positive cancer cells is currently unknown. Many studies have shown that tumor cells tend to endocytose nanostructures of appropriate size [34] through mechanisms such as macropinocytosis, clathrin-mediated endocytosis, and caveolae-mediated endocytosis [35–37]. However, because Apt-Td was negatively charged due to its DNA scaffold, there should be a repulsive force between Apt-Td and the cells, which were also negatively charged. This repulsive force may partially explain why limited Apt-Td entered the MUC1-negative control cells (Figure 5). For MUC1-positive cancer cells, however, the binding between the aptamer and the cell may overcome the repulsive force and pull together the nanostructure and the cell. As a result, the process of endocytosis was facilitated, and more drugs were delivered into the cell (Figure 5). Although this hypothesis may partially explain the MUC1-targeting behavior of Apt-Td, extensive future studies are needed to reveal the detailed mechanisms of Apt-Td cellular uptake.

Previous studies have shown that MUC1 aptamer can serve as an effective tumor-targeting ligand for selective delivery of anticancer drugs to tumor cells. Ferreira et al. used a MUC1 aptamer for targeted photodynamic therapy and demonstrated a 500-fold increase of light-induced toxicity to cancer cells [17]. Hu et al. showed that free MUC1 aptamers could be loaded with doxorubicin for selective drug delivery to MUC1-positive cancer cells *in vitro* [21]. In another study, Yu et al. showed that a MUC1 aptamer promoted the targeted delivery of paclitaxel encapsulated in a PLGA nanoparticle to MUC1-positive cancer cells [22]. In agreement with these studies, here we also observed that MUC1 aptamer could significantly enhance the tumor-targeted delivery of doxorubicin carried by DNA tetrahedron.

Compared to the aforementioned MUC1-targeted therapeutic systems, the present study design has some unique features. Although free MUC1 aptamer may carry doxorubicin for targeted delivery [21], an aptamer *per se* usually has a very small Dox-loading capacity. Moreover, an aptamer has an extremely small size and is prone to renal clearance *in vivo*, resulting in a very short half-life that significantly limits its value as a practical drug carrier. Prior study by Kim et al showed that DNA Td could carry a higher load of doxorubicin (26 per Td) for drug delivery [26]. In this study, a tumor-targeting aptamer was coupled to a DNA Td to form the Apt-Td, which not only had a significantly higher drug-loading capacity (25-fold) compared to a free MUC1 aptamer (Figure 4), but also selectively delivered the drug to MUC1-positive cancer cells. Additionally, Apt-Td had an average size of 12 nm, which would markedly decrease the chance for being cleared through the kidneys [38]. Moreover, our results showed for the first time that a MUC1 aptamer could be coupled with a DNA tetrahedron in a self-assembled way and that doxorubicin could be easily loaded into Apt-Td in a one-step reaction with high efficiency. This self-assembled approach significantly simplified the procedure for preparation of the MUC1-targeted drug carrier, compared with that for encapsulating the drug with polymer-based nanoparticles [22]. These features of Apt-Td may facilitate its preclinical and clinical development as a MUC1-targeted drug delivery system.

MUC1 is considered a high-value molecular target for cancer treatment because it is widely expressed in most adenocarcinomas, including cancers of breast, lung, colon, prostate, stomach, pancreas, and ovary. Moreover, the expression of MUC1 in tumors is overexpressed than that in normal tissue. This discrepancy in expression makes it possible for MUC1-targeted therapeutic systems to enrich anticancer drugs in tumors. To date, there is a paucity of research on the development of MUC1-targeted carrier systems for the delivery of cytotoxic drugs. In this study, we designed a new MUC1-targeted carrier system that can be easily constructed in a self-assembled manner. With further development, this simple system of Apt-Td may potentially be used to treat many types of tumors that overexpress MUC1, reducing systemic toxicity, and enhancing the efficacy of doxorubicin. Nevertheless, the current study is merely a proof-of-concept study. Extensive future research work is still required to develop Apt-Td into a practical MUC1-targeted drug carrier for clinical applications. Although Apt-Td showed MUC1-targeting capability *in vitro*, future studies are still warranted to demonstrate its functionality *in vivo*. To achieve this goal, extensive chemical modifications of Apt-Td is necessary to improve the nuclease-resistance of the nanostructure in blood. Moreover, in-depth evaluations of the pharmacodynamics, pharmacokinetics, and toxicological features of Apt-Td will also need to be addressed in future research.

In conclusion, a novel complex of MUC1 aptamer and DNA tetrahedron was constructed in a self-assembled way. The Apt-Td could selectively deliver doxorubicin into the MUC1-positive breast cancer cells *in vitro*. Such a system, with future development, may have application potentials for targeted treatment of MUC1-expressing tumors.

## MATERIALS AND METHODS

### Materials

The MUC1 aptamer (Apt), which was selected by Ferreira et al. (5′-GCAGTTGATCCTTTGGATACCCTGG-3′) was utilized for this study [25]. A modified MUC1 aptamer (Apt-tail) (5′-TTCCCTTCCTTCTCTCTTCCTCTCTCGCAGTTGATCCTTTGGATACCCTG-3′) was also synthesized. Some aptamers were labeled with 5′-FAM as needed. The DNA tetrahedron was comprised of four DNA single strands, strand A (5′-ACATTCCTAAGTCTGAAACATTACAGCTTGCTACACGAGAAGAGCCGCCATAGTA-3′), strand B (5′-TATCACCAGGCAGTTGACAGTGTAGCAAGCTGTAATAGATGCGAGGGTCCAATAC-3′), strand C (5′-TCAACTGCCTGGTGATAAAACGACACTACGTGGGAATCTACTATGGCGGCTCTTC3′), and strand D (5′-TTCAGACTTAGGAATGTGCTTCCCACGTAGTGTCGTTTGTATTGGACCCTCGCAT-3′). A sticky end was extended from strand A to be hybridized with the Apt-tail (5′-AGGAAGAGAGAAGGAAGGGAATTTTTACATTCCTAAGTCTGAAACATTACAGCTTGCTACACGAGAAGAGCCGCCATAGTA-3′). All DNAs were synthesized by Invitrogen (Shanghai, China).

### Cell culture

The MUC1-positive cell lines (MCF-7, A549 and HT-29) and MUC1-negative cell lines (MDA-MB-231, HepG2 and L02) were obtained from the Cell Center of Chinese Academy of Medical Sciences (Beijing, China). MCF-7, A549, HT-29, HepG2 and L02 cells were cultured in DMEM medium and MDA-MB-231 cells were cultured in RPMI 1640 medium. The cell culture medium was purchased from Gibco. Both media were supplemented with 100 units/ml aqueous penicillin G, 100 mg/mL streptomycin, and 10% FBS at concentrations to allow 70% confluence in 24 hours.

### DNA Tetrahedron (Td) and Apt-Td preparation

Four single strand DNAs (A, B, C, D) were separately dissolved in TE buffer (10 mM Tris-HCl, 1 mM EDTA, pH 8.0), then mixed in TM buffer (20 mM Tris-HCl, 50 mM MgCl_2_, pH 8.0) at an equal molar ratio. The mixture was incubated at 90°C for two minutes, placed on ice for 5 minutes to rapidly cool the mixture, and then maintained at room temperature for 10 minutes to yield the DNA tetrahedron (Td). To form the aptamer-DNA tetrahedron (Apt-Td), the Apt-tail was added at an equal molar ratio with the Td and incubated at 37°C for 90 minutes.

### Characterization of nanoparticles

Agarose gel electrophoresis was applied to monitor the formation of the Td and Apt-Td. The gel was measured under UV light with the assistance of a DNA fluorescent dye DNA Green, which can outline double-stand DNA. The size and zeta-potential of the Td and Apt-Td were determined by dynamic light scattering (Malvern Zetasizer Nano ZS, UK). Ten pmol Td or Apt-Td were dissolved in 1.0 ml double-distilled water, and the particle size distributions were measured at a scattering angle of 90°. The intensity-weighted and zeta-potential mean value was recorded as the average of three measurements.

### Cellular binding of aptamers and Apt-Td

The cellular binding experiment was performed by flow cytometric (FCM) analysis. MCF-7, A549, HT-29, MDA-MB-231, HepG2, and L02 cells were gently scraped and washed with Hanks buffer twice. The cells were suspended in 200 μl of PBS, incubated with FAM-labeled random DNA, Apt, Apt-tail or Apt-Td separately at a concentration of 300 nM for 30 minutes, washed twice with Hanks buffer, and then resuspended in 200 μl PBS. The FCM analysis was performed to examine the binding of random DNA, Apt, Apt-tail, or Apt-Td to both cell lines.

### Drug-loading capacities of Td and Apt-Td

The fixed concentration of Dox (3 nM) was incubated with Apt, Td, or Apt-Td for one hour in a 96-well black plate at various carrier/Dox molar ratios. The fluorescence spectrum of doxorubicin was examined by a Synergy4 analyzer (λEx = 488 nm, λEm = 520–720 nm).

### Cellular uptake studies

The cellular uptake of Dox was studied by confocal microscopy (Perkin Elmer Ultraview, US). Cells were allowed to adhere to a glass coverslip for 24 hours. The cells were incubated with free Dox, Dox-loaded Td (Td-Dox), and Dox-loaded Apt-Td (Apt-Td-Dox) at an equivalent dose of Dox at 2 μM for 1.5 hours at 37°C and washed twice with Hanks buffer. The cells were then fixed with 4% formaldehyde for 10 minutes at 4°C, washed twice with Hanks buffer. Ten microliters of DAPI was added to the slide, and then a glass coverslip with cells was sealed and stained for 5 minutes. Confocal fluorescence scanning microscopy was used to evaluate cell fluorescence.

For flow cytometric analysis, cells were scraped off from the culture bottle and washed twice with Hanks buffer. The cells were incubated with free Dox, Td-Dox, or Apt-Td-Dox at an equivalent dose of Dox at 2 μM for 1.5 hours at 37°C, and washed twice with Hanks buffer. The cells were then fixed with 4% formaldehyde for 10 minutes and analyzed by flow cytometry.

Co-culture experiments were performed to evaluate the targeting specificity of Apt-Td-Dox. MDA-MB-231 cells were incubated with the fluorescent dye CFSE for 10 minutes at 37°C, washed by Hanks buffer thrice, and suspended in DMEM medium. The MDA-MB-231 cells were co-cultured on a cover glass together with MCF-7 cells that were not stained with CFSE. After 24 hours, the cells were washed twice with Hanks buffer, incubated with free Dox, Td-Dox, or Apt-Td-Dox at an equivalent dose of Dox of 2 μM for 1.5 hours at 37°C, washed twice, and fixed with 4% formaldehyde for 10 minutes at 4°C. The cover glass with cells was sealed and evaluated by confocal fluorescence scanning microscopy.

### *In vitro* cytotoxicity

To evaluate the cytotoxic effects of Apt-Td-Dox against MUC1-positve and MUC1-negative cells, both cell lines were grown in 96-well plates. The cells were treated with free Dox, Td-Dox, or Apt-Td-Dox. MCF-7 and MDA-MB-231 cells were co-cultured with the respective substances for each treatment group at an equivalent dose of Dox at 2 μM for two hours at 37°C. The cells were washed with Hanks buffer three times and cultured for an additional 48 hours. After these procedures, an MTS assay (Promega, US) was used to determine the cell viability per standard protocol outlined by the manufacture.

### Internalization time course of Apt-Td-Dox

To investigate the internalization speed of Apt-Td-Dox into the cells, MCF-7 and MDA-MB-231 cells were grown in 6-well plates, and incubated at 37°C with free Dox or Apt-Td-Dox for 0.25, 0.5, 0.75, 1, 1.5, 2, 4, 6, and 8 hours, respectively. The cells were gently scraped and washed with Hanks buffer twice, fixed with 4% formaldehyde for 10 minutes, and analyzed by flow cytometry. The internalization time course was plotted by the GraphPad Prism 5 software.

### Evaluation of IC50

To evaluate the IC50 of Apt-Td-Dox against MUC1-positve and MUC1-negative cells, MCF-7 and MDA-MB-231 cells were grown in 96-well plates and treated with either free Dox or Apt-Td-Dox for two hours at 37°C. MCF-7 cells were incubated with treatments equivalent to Dox of 0.01, 0.02, 0.2, 1, 2, 5, 10, and 20 μM, respectively. MDA-MB-231 cells were incubated with treatments equivalent to Dox of 0.01, 1, 2, 10, 20, 25, 30, and 35 μM, respectively. The cells were washed with Hanks buffer for three times and cultured for additional 48 hours. MTS assay was used to determine the cell viability according to the protocol as outlined by the manufacture (Promega). The data were collected, and the IC50 curves were created by GraphPad Prism 5 software.

### Statistical analysis

Statistical analysis was performed using Statistical Analysis System (SAS, Version 9.2). One-way ANOVA with Fisher's least significant difference (LSD) post hoc comparisons at 99% confidence interval was used for statistical comparisons. All data are presented as a mean value with its standard deviation indicated (mean ± SD).
